# Association between magnesium in drinking water and atrial fibrillation incidence: a nationwide population-based cohort study, 2002–2015

**DOI:** 10.1186/s12940-021-00813-z

**Published:** 2021-12-15

**Authors:** Kirstine Wodschow, Cristina M. Villanueva, Mogens Lytken Larsen, Gunnar Gislason, Jörg Schullehner, Birgitte Hansen, Annette Kjær Ersbøll

**Affiliations:** 1 National Institute of Public Health, University of Southern Denmark, Studiestræde 6, 1455 Copenhagen C, Denmark; 2 ISGlobal, Campus Mar, Dr. Aiguader, 88, 08003 Barcelona, Spain; 3 CIBER Epidemiología y Salud Pública (CIBERESP), Av. Monforte de Lemos 3-5. Pabellón 11. Planta 0, 28029 Madrid, Spain; 4 Universitat Pompeu Fabra (UPF), Plaça de la Mercè, 10-12, 08002 Barcelona, Spain; 5 IMIM (Hospital del Mar Medical Research Institute), Dr. Aiguader, 88, 08003 Barcelona, Spain; 6 Department of Clinical Medicine, Aalborg University, Søndre Skovvej15, 9000 Aalborg, Denmark; 7 Department of Cardiology, The Cardiovascular Research Centre, Copenhagen University Hospital Herlev and Gentofte, Gentofte Hospitalsvej 1, 2900 Hellerup, Denmark; 8 Faculty of Health and Medical Sciences, University of Copenhagen, Blegdamsvej 3B, 2200 Copenhagen N, Denmark; 9 The Danish Heart Foundation, Vognmagergade 7, 3. sal, 1120 Copenhagen C, Denmark; 10 Geological Survey of Denmark and Greenland, GEUS Department of Groundwater and Quaternary Geology Mapping, C.F. Moellers Allé 8, Bygning 1110, 8000 Aarhus C, Denmark; 11 Department of Public Health –Research Unit for Environment, Work and Health, Aarhus University, Bartholins Allé 2, 8000 Aarhus C, Denmark

**Keywords:** Atrial fibrillation, Magnesium level, Drinking water, Cohort, Registers

## Abstract

**Background:**

Atrial fibrillation (AF) is a common heart rhythm disorder and a risk factor of adverse cardiovascular diseases. Established causes do not fully explain the risk of AF and unexplained risk factors might be related to the environment, e.g. magnesium in drinking water. Low magnesium levels in drinking water might be associated with higher risk of cardiovascular diseases including AF. With detailed individual data from nationwide registries and long-term magnesium exposure time series, we had a unique opportunity to investigate the association between magnesium in drinking water and AF.

**Objective:**

We evaluated the association between magnesium concentration in drinking water and AF risk.

**Methods:**

A nationwide register-based cohort study (2002–2015) was used including individuals aged ≥30 years. Addresses were linked with water supply areas (*n* = 2418) to obtain time-varying drinking water magnesium exposure at each address. Five exposure groups were defined based on a 5-year rolling time-weighted average magnesium concentration. AF incidence rate ratios (IRRs) between exposure groups were calculated using a Poisson regression of incidence rates, adjusted for sex, age, and socioeconomic position. Robustness of results was investigated with different exposure definitions.

**Results:**

The study included 4,264,809 individuals (44,731,694 person-years) whereof 222,998 experienced an incident AF. Magnesium exposure ranged from 0.5 to 62.0 mg/L (mean = 13.9 mg/L). Estimated IRR (95% CI) compared to the referent exposure group (< 5 mg/L) was 0.98 (0.97–1.00) for the second lowest exposure group (5–10 mg/L), and 1.07 (1.05–1.08) for the two highest exposure groups (15–62 mg/L). Strongest positive associations were observed among those aged ≥80 years and with lowest education group. An inverse association was found among individuals with highest education group.

**Conclusion:**

There might be a small beneficial effect on AF of an increase in magnesium level in drinking water up to 10 mg/L, though an overall positive association was observed. The unexpected positive association and different associations observed for subgroups suggest a potential influence of unaccounted factors, particularly in vulnerable populations. Future research on magnesium in drinking water and cardiovascular diseases needs to focus on contextual risk factors, especially those potentially correlating with magnesium in drinking water.

**Supplementary Information:**

The online version contains supplementary material available at 10.1186/s12940-021-00813-z.

## Background

Atrial fibrillation (AF) is a common heart rhythm disorder affecting more than 34 million people (estimated global prevalence, 2010 [1]) and the life-time risk of AF is approximately 1 out of 6 [2]. AF is a risk factor of adverse cardiovascular outcomes, including stroke and heart failure [3]. Specifically, AF is associated with a five-fold higher risk of stroke [4], in addition to a reduced quality of life [5]. Both prevalence and incidence of AF are increasing worldwide [1, 6], and established risk factors include age, male sex, hypertension, valvular heart disease, left ventricular systolic dysfunction, obesity and alcohol consumption [7]. Established risk factors may explain approximately 56% AF risk [8] and research in risk factors is therefore needed. Geographical variations exist in AF risk [9] and recently, more research in risk factors related to the environment is seen, such as air pollution [10, 11], noise [12] and deprivation [13].

Water hardness, calcium and specifically magnesium have been inversely linked to a reduced risk of cardiovascular diseases (CVDs) [14–17]. In countries, with a decentralised water supply structure, inorganic chemical composition of groundwater based drinking water may differ between waterworks and magnesium in drinking water can therefore represent a neighbourhood risk factor. The association between low concentrations of magnesium in drinking water and risk of CVD has been studied for several decades, with diverging evidence. An inverse association between magnesium concentrations and CVD related mortality has been found [15, 18, 19]. However, other studies found no such association [20–22] and even a positive association has been found between incidence of coronary heart disease and magnesium in drinking water in a group of men [23] and a group of women [20]. Magnesium is regarded as having a positive effect on CVD, if any effect. A too high body magnesium level is rare among healthy persons, however, it can be seen in persons with a combination of decreased renal function and a high magnesium intake from e.g. supplements [24]. To our knowledge the association between incident AF and magnesium in drinking water has not yet been investigated. An association between low serum magnesium and increased risk of AF has been found [25], but no association was found for dietary magnesium and AF [26]. The underlying plausible biological mechanism between a low magnesium intake and AF might include inflammation, oxidative stress and electrical remodelling, as reviewed for CVDs by Liu & Dudley Jr. [27].

Due to lack of knowledge, the World Health Organization (WHO) [28] has not recommended any magnesium minimum concentrations in their guidelines for drinking water quality. They state that further studies are needed, including studies of different outcomes in combination with mortality [29]. The need for further studies may be even more relevant in light of the widely use of residential water softeners [30] and e.g. increasing use of desalination of sea water as a solution to water scarcity. During desalination of seawater magnesium concentration in drinking water may be reduced, unless water is remineralised after desalination.

The magnesium level in Danish drinking water resembles the mean of European tap water (9.6 mg/L) (Euro Geo Surveys, 2010 in [31]), however large variations from less than 5 mg/L and > 200 mg/L are expected in groundwater based drinking water systems [29]. Drinking water in Denmark is entirely from groundwater sources [32], and use of bottled drinking water is low (21 L per year per capita in 2018) [33]. Mean and median magnesium concentration in drinking water are 12.1 mg/L and 9.8 mg/L, respectively, and ranges from 2.5–35 mg/L, 2.5 and 97.5 percentiles, respectively [34]. Magnesium in drinking water can therefore be an important daily magnesium source.

Based on nationwide population-based individual-level register data including adults aged ≥30 years, we examined the association between residential drinking water magnesium level and the incidence of AF. The objective of the study was three-fold: 1) estimate the exposure to magnesium in drinking water for the adult population in Denmark, 2) examine if exposure to low magnesium level in drinking water was associated with an increased risk of AF, when accounting for sex, age, socioeconomic position and calendar year, and 3) investigate if the results were consistent by carrying out sensitivity analyses with different exposure definitions and stratified analyses.

## Methods

### Study design and population

**Fig. 1:**
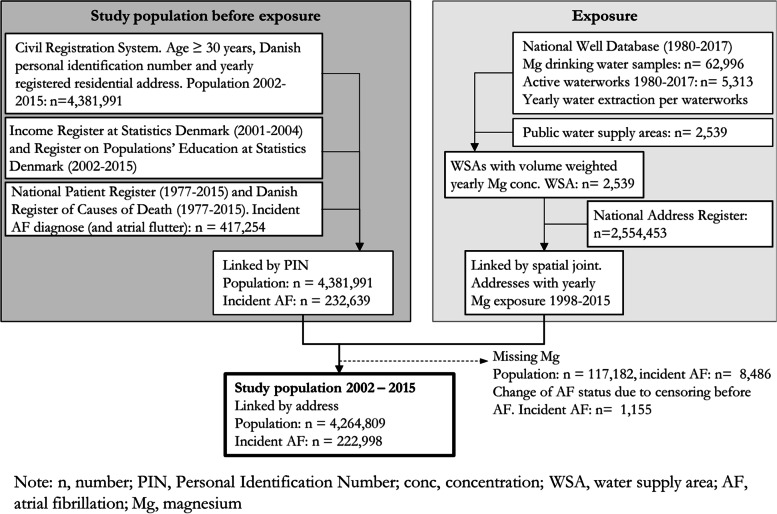
Flow chart of register linkage, creation of exposure and the study population

We designed a national open cohort study including the Danish population at age 30 years or more at inclusion in the study period 2002–2015. Danish national registers used in the present study were linked at an individual level by the unique personal identification number (PIN), given to all Danish residents at birth or at immigration [35]. Linkage of registers and linkage of exposure to the cohort is shown in Fig. 1. Individuals were included at study start (1/1–2002) if born before the 1st of January 1972 and otherwise at their 30th birthday. Furthermore, individuals had no former AF diagnosis, a Danish personal identification number, no missing residential address between 1987 and up to date of entry and were living at an address where magnesium exposure was assigned. Individuals were followed until the first of the following events: incident AF, death, unknown magnesium exposure, or no registered address in Denmark. Re-entry to the cohort was not allowed.

### Atrial fibrillation

Incident AF was defined as a person’s first-ever occurring AF or the less common atrial flutter diagnosis (diagnoses registered since 1977) registered in the National Patient Register [36] or The National Register of Causes of Death [37] in the study period 2002–2015. To identify incident AF in the study period, all individuals with an AF diagnosis before inclusion in the study were excluded. Consistent with the Framingham Heart Study [38] both AF and atrial flutter were included and defined according to the International Classification of Diseases (ICD), 8th revision (ICD-8) from 1977 until 1993 and 10th revision (ICD-10) hereafter. ICD-8 codes included 427.93, 427.94 and 427.9 and code I48 in ICD-10. The positive predictive value for both AF and atrial flutter has been estimated as high (92.6%) in the National Patient Register [39].

### Magnesium exposure assessment

We analysed routine monitoring data of magnesium concentration in drinking water measured at the waterworks and registered in the national well database “Jupiter” [40]. We estimated yearly mean magnesium concentrations in drinking water for each public water supply area in 1998–2015. A waterworks may supply several water supply areas and each water supply area may be supplied by multiple waterworks. Magnesium concentration at each water supply area was previously calculated as a yearly mean weighted by the amount of water abstracted at each waterworks within each water supply area (see Fig. 1 for linkage of data) [34]. A large variation in the number of measurements per water supply area exist and linear interpolation was used to estimate magnesium concentration for years with missing data. Each water supply area in Denmark has previously been categorized in six categories according to temporal variation in magnesium concentration for the period 1980–2017 [34]. In the present study we applied the categorisation by dividing the water supply areas as having either a stable temporal magnesium concentration level or unstable. A stable magnesium level includes water supply areas where the magnesium concentration earlier has been defined as constant, increasing/decreasing or as having two different constant concentration levels separated in time [34]. Unstable water supply areas include e.g. those water supply areas where the magnesium level is fluctuating or there are too few magnesium concentration measurements to see a trend.

Magnesium concentration (1998–2015) was assigned to addresses registered in the National Address Database [41] by spatial joint (Fig. 1). Afterwards, yearly magnesium exposure was assigned to each individual in the cohort, according to residential address for each year in the period 2002–2015. No magnesium exposure was assigned to individuals for years lived at an address with a private well within 50 m of registered geographic coordinates of the address. Hence, individuals who had only address registrations near a private well were excluded from the cohort. The magnesium concentration of drinking water in private wells is not included in the regulations regarding mandatory testing of water quality [42]. Therefore, the number of measurements of the magnesium concentration at private wells is very sparse.

To account for a potential long-term effect of magnesium in drinking water a five-year rolling time-weighted average (TWA) magnesium exposure was calculated for each individual each year in the cohort. The five-year rolling TWA was calculated for each individual as the average of the magnesium concentration in the drinking water the given year at the given address and the magnesium concentration at the residential addresses the previous 4 years. The concentration was weighted according to days lived each year at the address. Thereby, the five-year rolling TWA magnesium exposure reflects changes in magnesium concentration in drinking water when e.g. an individual changed residential address from an area with a high magnesium concentration in the drinking water to an area with a low magnesium concentration. If magnesium concentration was missing for less than 5 years for an individual, the yearly mean magnesium concentration was calculated using the number of years with measured magnesium. If magnesium concentration was missing for more than 5 years, the magnesium exposure could not be calculated for that individual for the given period. Finally, five-year rolling TWA magnesium exposure for each individual was categorized in 5 exposure groups (0.1–5, > 5–10, > 10–15, > 15–20 and > 20 mg/L).

### Covariates

Individual data on sex, age, cohabitation and calendar year were drawn from the Danish Civil Registration System [35]. Age was categorized in 5-year age groups (30–34, 35–39, 40–44, …, 85–89, ≥90 years). Cohabitation was defined as married/living with a partner or living alone. Calendar years were categorized in seven two-years bands starting from 2002. Family equivalent individual income was drawn from the National Income Register [43] and categorized in quintiles within groups of sex, calendar year and above/below 65 years (e.g. individuals in the lowest income quintile in the group of men below 65 years in 2010 were in the same income category as individuals in the lowest income quintile in the group of women above 65 years in 2015). Education was categorized as short (≤9 years), middle (10–12 years) or long (≥13 years) education using data drawn from the National Education Register [44]. When education level was missing for a year, the educational level assigned to previously years and otherwise the following years (if previous years were missing) was assigned. If education was registered as “unknown” (8.3% of the study population) the lowest education group was assigned, since most of the individuals with missing educational level belonged to the lowest income group.

### Statistical analyses

To investigate the association between magnesium in drinking water and AF, we used a Poisson regression with number of incident AF as outcome and logarithmic transformation of person-years as the offset (piecewise exponential model). Person-years were split according to calendar year and age in 5-year intervals. Magnesium exposure groups, sex, age, calendar year, income, education and cohabitation were included in the model as fixed effects. The association was presented as incidence rate ratio (IRR) between the 5 exposure groups with a corresponding 95% confidence interval (95% CI). Initially, the association was analysed with a crude model (unadjusted), a semi-adjusted model (adjusted for age, sex and calendar year) and a fully adjusted model with further adjustment for socioeconomic position (education, income and cohabitation). A trend analysis was performed to evaluate a possible doses-respond association. The mean magnesium concentration within each of the 5 exposure groups was included as a continuous variable in the fully adjusted model. Furthermore, we stratified the fully adjusted model by sex, age and education, to investigate for differences in association between the groups. Tests of interaction between magnesium and sex, age and education were done by including an interaction term in the fully adjusted model and performing likelihood ratio tests.

The robustness of the results was investigated by performing four sensitivity analyses. First, the five-year rolling TWA magnesium concentration was changed to a one-year and a two-year rolling TWA, respectively. Secondly, only person-years in the first exposure group for each individual were included. This means that the first time an individual changed exposure group; the person-years were censored for that individual. Thirdly, magnesium exposure was divided in 7 groups (0.1–5, > 5–7.5, > 7.5–10, > 10–12.5, > 12.5–15, > 15–20, > 20 mg/L), in order to investigate risk differences for the lowest magnesium exposure levels. Finally, two analyses were performed on selected water supply areas. In the first analysis only individuals who were supplied by water from a water supply area with at least one water sample for every second year in 2002–2015 were included. In the second analysis individuals were included if they were supplied by water from a water supply area where the magnesium concentration has been categorized as having a stable temporal magnesium concentration level. One post-hoc analysis was performed to investigate the influence of regional structured risk factors for AF. In the post-hoc analysis, the fully adjusted model was repeated on a study population restricted to only include individuals with an address within the Region of Southern Denmark.

Visualization of water supply areas and spatial joint between each address and water supply areas was done in Quantum GIS version 3.2.1. (www.qgis.org). All data management and analyses were performed in STATA statistical software version15.1 (Stata College Station, TX).

## Results

The final study population included 4,264,809 individuals (44,731,694 person-years) whereof 222,998 were diagnosed with an incident AF during the study period 2002–2015 (Fig. 1) (incidence rate, IR at 498.5 per 100,000 person-years). No income could be assigned for 1173 individuals (including 16 incident AF). A total of 117,182 individuals (including 8486 incident AF) were excluded from the initial population (2002–2015) since no magnesium exposure could be assigned, due to either assumed supplied by private well (51%), no magnesium measurement at the water supply area (38%), no link between addresses and population data (9%) or a combination of the three (2%). In total, 2418 water supply areas were included with at least one magnesium water sample during 2002–2015. Magnesium exposure ranged from 0.1–62.0 mg/L (mean = 13.9 mg/L; median = 12.0 mg/L). On average, individuals were followed for 10.7 years and for approximately 90% of the individuals it was possible to assign a yearly magnesium exposure each year. A total of 2,670,299 were at least once supplied by water from a water supply area with at least one magnesium sample per every second year (480 water supply areas) and 548,676 were supplied by water from a water supply area categorized as stable (1680 water supply areas). In the post-hoc analysis 542,602 individuals were included (560 water supply areas).

Baseline characteristics at date of inclusion according to the five magnesium exposure groups are presented in Table 1. The distribution of the study population between magnesium exposure groups is uneven, ranging from about 10% of the individuals in the lowest and second highest exposure groups and up to 29% in the highest exposure group. The majority of the individuals entered the cohort in 2002–2003 (76%), were 30–59 years (77%), with 10–12 years education (43%) and were married or living with a partner (67%). Due to the nature of how income groups were defined, the distribution in income groups was fairly even, with a slightly higher percentage in the lowest income group (25%). The magnesium exposure distribution for each characteristic was similar to the overall distribution, with the exception of cohabitation and education. More individuals living alone, and with ≥13 years education were in the highest exposure group.

**Table 1:** Characteristics of the cohort at inclusion by magnesium concentration at residential address

Characteristic	Category	Individuals *n* (%)	Individuals (%) for each magnesium group (mg/L)				
			1 (0.1–5)	2 (> 5–10)	3 (> 10–15)	4 (> 15–20)	5 (> 20–62)
Overall		4,264,809 (100)	10.6	28.3	20.4	12.2	28.6
Sex	*Female*	2,183,300 (51.2)	10.5	28.3	20.2	12.3	28.7
	*Male*	2,081,509 (48.8)	10.7	28.4	20.5	12.0	28.4
Age	*30–59*	3,269,858 (76.7)	10.6	27.8	20.5	11.8	29.3
	*60–69*	475,514 (11.1)	10.8	30.6	20.3	13.5	24.9
	*70–79*	327,352 (7.7)	10.6	30.4	19.6	13.5	25.9
	*≥ 80*	192,085 (4.5)	10.1	29.3	19.0	12.6	29.0
Civil status	*Cohabitating*	2,850,546 (66.8)	11.5	29.9	20.6	12.3	25.7
	*Living alone*	1,414,263 (33.2)	8.7	25.1	20.0	11.8	34.4
Education	*≤ 9 years*	1,576,835 (37)	11.6	29.6	20.0	12.0	26.8
	*10–12 years*	1,812,707 (42.5)	11.0	29.0	20.8	12.3	26.8
	*≥ 13 years*	875,267 (20.5)	7.9	24.6	20.1	12.0	35.4
Income	*Lowest*	1,054,367 (24.7)	9.7	26.7	20.8	11.1	31.7
	*Second lowest*	867,631 (20.3)	12.0	31.0	21.4	11.3	24.3
	*Middle*	831,798 (19.5)	11.7	30.1	20.8	11.8	25.6
	*Second highest*	783,979 (18.4)	10.7	28.4	20.2	12.5	28.2
	*Highest*	727,034 (17)	8.7	25.5	18.1	14.7	33.0
Calendar year	*2002–2003*	3,232,498 (75.8)	10.4	29.5	20.2	12.3	27.7
	*2004–2005*	200,827 (4.7)	13.6	28.8	20.1	9.2	28.2
	*2006–2007*	175,448 (4.1)	10.8	25.2	22.9	14.9	26.3
	*2008–2009*	167,241 (3.9)	11.2	25.3	20.8	8.9	33.8
	*2010–2011*	170,972 (4)	9.3	19.8	20.7	18.8	31.3
	*2012–2013*	155,102 (3.6)	10.8	25.4	20.6	9.3	33.9
	*2014–2015*	162,721 (3.8)	10.6	23.8	20.6	9.2	35.7
Water supply area category	*Stable*	1,571,831 (36.9)	17.0	38.6	24.1	11.4	8.9
	*Unstable*	2,680,928 (62.9)	6.8	22.3	18.2	12.6	40.1
	*Missing*	12,050 (0.3)	9.3	28.1	21.3	13.1	28.2

Table 2 shows the results of the association between magnesium exposure and incidence rate (IR) of AF for the crude (unadjusted), semi-adjusted (adjusted for age, sex and calendar year) and fully adjusted model (further adjusted for socioeconomic position). In the fully adjusted model, an IRR of 0.98 (95% confidence interval (CI): 0.97–1.00)) was estimated between the second lowest (5–10 mg/L) and lowest exposure group (0.1–5 mg/L). On the contrary, an IRR of 1.07 (95% CI: 1.05–1.08) was estimated for the two highest exposure groups (15–62 mg/L) compared to the lowest exposure group. The trend analysis showed a small positive dose-response association with an IRR of 1.043 for a 10 mg/L increase in magnesium exposure (95% CI: 1.038–1.049) in the fully adjusted model. The largest change in IRR was seen between the crude and semi-adjusted model in the highest magnesium exposure group compared to the lowest (IRR changed from 0.98 to 1.05).

**Table 2:** Main results of the association between magnesium in drinking water as five residential exposure groups and incidence of atrial fibrillation

Magnesium exposure group	PY	Incident AF	IR per 100.000 PY	IRR (95% CI)		
				Crude^a^	Semi-adjusted^b^	Fully adjusted^c^
1: 0.1–5 mg/L	4,915,009	24,671	502	1.00 (ref)	1.00 (ref)	1.00 (ref)
2: > 5–10 mg/L	12,307,633	60,468	491	0.98 (0.96; 1.19)	0.98 (0.96; 0.99)	0.98 (0.97; 1.00)
3: > 10–15 mg/L	10,063,416	49,749	494	0.98 (0.97; 1.00)	1.00 (0.99; 1.02)	1.01 (0.99; 1.02)
4: > 15–20 mg/L	5,856,071	30,940	528	1.05 (1.04; 1.07)	1.05 (1.03; 1.06)	1.07 (1.05; 1.08)
5: > 20–60 mg/L	11,589,566	57,170	493	0.98 (0.97; 1.00)	1.05 (1.04; 1.07)	1.07 (1.05; 1.08)

Note: *PY* Person years, *AF* Atrial fibrillation, *IR* Incidence rate, *IRR* incidence rate ratio, *CI* Confidence interval

^a^No adjustment

^b^Adjusted for age, sex, and calendar year

^c^Adjusted for age, sex, calendar year, cohabitation, education and income

**Fig. 2:**
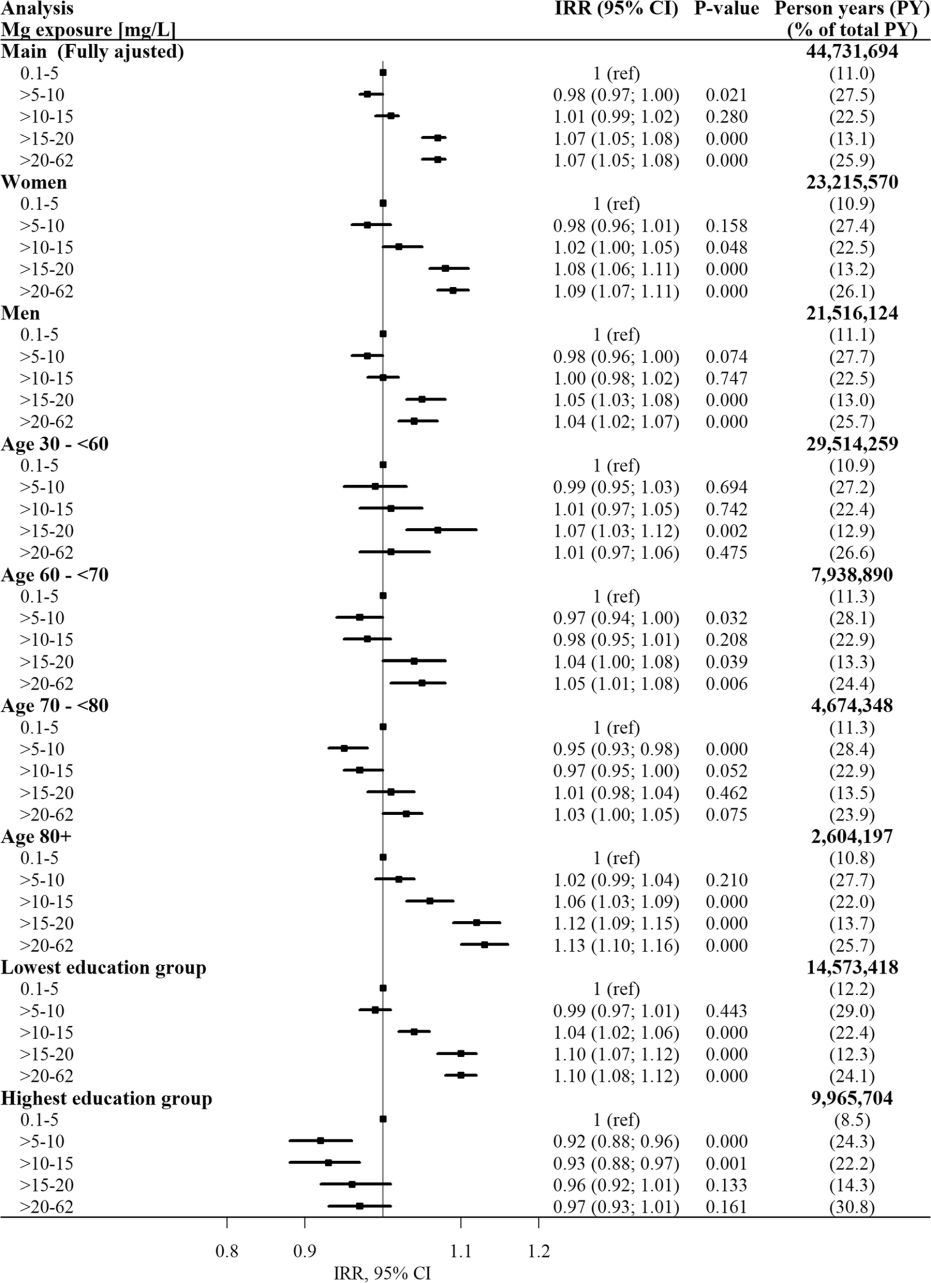
Incidence rate ratios (IRRs) of atrial fibrillation and confidence intervals (CI) presented in a forest plot for both the fully adjusted model (adjusted for age, sex and socioeconomic position) and stratified analyses. The lowest magnesium (Mg) exposure group is the reference group in all analyses

Results of the stratified analyses of the fully adjusted model are shown in Fig. 2 including the fully adjusted main analysis. Interaction was observed between magnesium and both age (*p*-value < 0.0001) and education (p-value < 0.0001). No association was observed between magnesium and sex (p-value = 0.0533). The estimated IRRs for age groups 30–60, 60–70 and 70–80 years were similar to the estimated IRRs in the fully adjusted model. For the oldest age group, the estimated IRRs were considerable higher than for the other three age groups, and a tendency to increasing risk of incident AF with exposure to increasing magnesium concentration was found, though the result was statistically non-significant for exposure group 2. When stratified by sex, the results showed a very similar exposure-response pattern for men and women. When only individuals in the highest education group were included, the IR was lower in the four highest exposure groups (concentration > 5 m/L) compared to the lowest exposure group, though the results were statistically insignificant for the two highest exposure groups (Fig. 2).

**Fig. 3:**
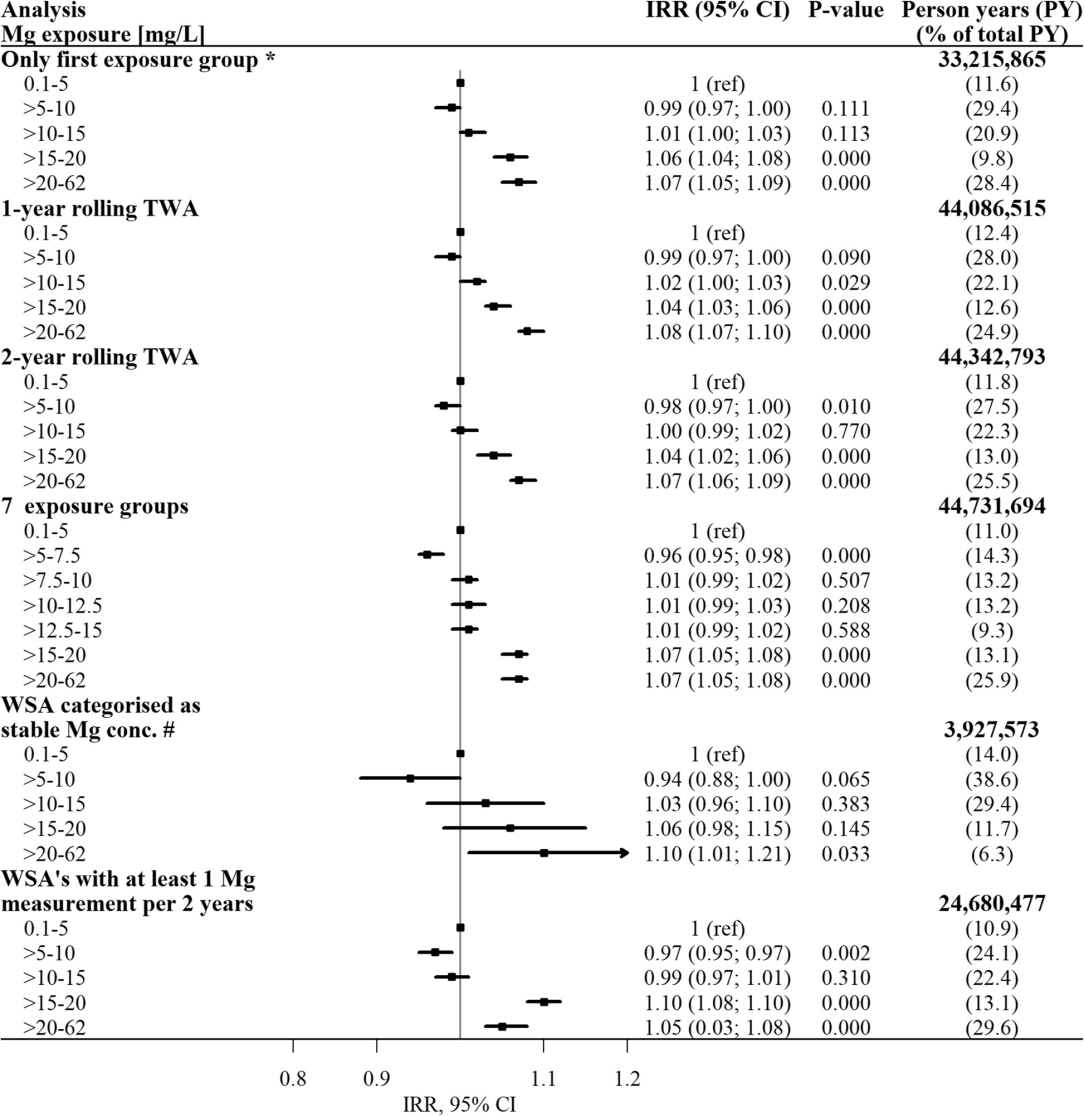
Incidence rate ratios (IRRs) of atrial fibrillation and confidence intervals (CI) presented in forest plots for the sensitivity analyses. The lowest magnesium (Mg) exposure group is the reference group in all analyses. All analyses are adjusted for age, sex and socioeconomic position

Figure 3 shows the results of the sensitivity analyses. Changing the 5-year rolling TWA to a one-year and two-year rolling TWA did not result in any substantial change in the IRRs. When restricting the analysis to only include person-years for each individual until first change of exposure group, we found similar results as for the main analyses. When dividing magnesium exposure into seven groups, we found an IRR of 0.96 (95% CI: 0.95–0.98) between the second exposure group (> 5–7.5 mg/L) and the lowest exposure group (0.1–5 mg/L) (Fig. 3). When only water supply areas with at least one water sample for every second year were included, the overall association between magnesium in drinking water and incident AF resembled the main results. The same overall association was found when only water supply areas defined as having a stable magnesium concentration were included. However, a much wider confidence interval was observed (Fig. 3). In the post-hoc analysis where only the Region of Southern Denmark was included, a lower risk of AF was observed for an exposure of 5–15 mg/L and > 20 mg/L, compared to the lowest exposure group (Additional file 1).

## Discussion

We examined the association between magnesium in drinking water and incident AF in Denmark. Though we found a wide contrast between low and high exposure to magnesium in drinking (ranging from 0.5 to 62 mg/L, mean = 13.9 mg/L) in this nationwide register-based study, we found no strong association between magnesium in drinking water and incident AF. AF incidence may be lower when exposed to a magnesium concentration of 5–10 mg/L in drinking water, compared to an exposure of 0.1–5 mg/L, though an overall small positive association was found. Higher incidence rates were observed among those aged 80 years and more, and among those in the lowest education group. An inverse association was found among those in the highest education group for all exposure groups compared to the lowest exposure group.

The estimated magnesium exposure interval (0.1–62.0 mg/L) and mean (13.9 mg/L) in the present study was wider compared to previous studies from the Nordic countries and the Netherlands on magnesium in drinking water and CVD, e.g. 1.17–5.87 mg/L (mean = 3.0 mg/L) in Finland [18], and 1.7–26.2 mg/L (mean 6.8 mg/L) in The Netherlands [20]. However, in England a wider exposure interval was estimated: 2–111 mg/L (mean 19 mg/L) [45].

Our findings of no clear inverse association support the earlier findings in studies of incident CVD, where no clear association between magnesium in drinking water and incident acute myocardial infarct [22, 46] and incident coronary heart disease [23] was found. A linear inverse dose-response effect up to about 8 mg/L [15] has been suggested for the association between magnesium in drinking water and cardiovascular mortality. However, our results suggest an unexpected positive association, though we do observe a lower risk of AF when exposed to 5–10 mg/L compared to less than 5 mg/L. Although when sub-dividing magnesium exposure into 7 groups, the lower risk of AF was observed only at 5–7.5 mg/L compared to less than 5 mg/L. A positive association has also been found by Morris, Walker [23], between coronary heart disease incidence and a two-fold increase in magnesium intake from tap water (Hazard ratio (HR) = 1.10; 95% CI: 1.00–1.20), adjusted for age and other cardiovascular risk factors. No dose-response relationship was found when dividing magnesium exposure into tertiles (magnesium concentration: < 5.3 mg/day, 5.3–14.1 mg/day, > 14.1 mg/day).

The recommended daily intake (RDI) of magnesium ranges from 280 to 420 mg [47]. Assuming an estimated daily tap water consumption of 1.85 L (estimated for the Swedish adult population, including boiled water [48]), the median magnesium intake in the highest exposure group accounts for around 10% of the RDI (9.9% for highest RDI - 14.9% for lowest RDI). In contrast, the coverage of daily magnesium intake through tap water was only 2% of the daily intake among adults aged 55–69 in the Netherlands [20], where no overall association was found between magnesium in tap water and mortality due to ischemic heart disease or stroke. Though their magnesium estimate was based on personal questionnaires and therefore expected to be more precise as a snapshot estimate, our magnesium estimate may be better as a long-term exposure estimate.

Both the identified interaction between magnesium and age and education, and the relatively different exposure-response pattern in the age stratified analyses, indicate that the association between increasing magnesium in drinking water and increasing AF incidence may be stronger for the oldest age group and lowest education group. However, since a too high body magnesium level is rare [24], it is probably something else that may explain the findings of a positive association. A possible explanation of the positive association might be due to different unaccounted causes of AF between the different age groups, e.g. hypertension. Furthermore, if the geographical variation in unaccounted risk resembles the geographical variation in magnesium in drinking water it could potentially influence the association. In addition, we found a lower risk of incident AF among individuals in the highest education group when exposed to > 5 mg/L as compared to 0.1–5 mg/L. In general, individuals with a high educational level have less comorbidities and better health behavior compared to individuals with a low educational level. Therefore, it suggests a small positive effect of magnesium in drinking water in the absence of strong individual risk factors and comorbidities (i.e. among individuals with a high educational level).

To our knowledge this is the first study on the association between magnesium in drinking water and incidence of AF and one of the first studies to use detailed and long-term registry data on both outcome, covariates, and magnesium exposure. The main strengths of the present study are the large study population, covering the entire Danish nation with administratively collected data selected independently of the aim of the study which limits selection bias [49] and a wide variability in magnesium exposure levels. In addition, AF is a well-defined diagnosis with a positive predictive value of AF at 92.6% in the National Patient register [39]. The geographical and temporal variations in magnesium in drinking water are well-studied [34], and the long time series of exposure and AF make it possible to follow changes in exposure category for each individual, by including changes in residential address. Furthermore, a decentralized water supply structure in Denmark results in relatively small water supply areas, which to some extent decreases the risk of misclassification.

Individuals with missing information on residential address prior to the study period were excluded (1987–2001), based on the possibility that incident AF may have occurred when living abroad. Since AF can be a less severe disease, it is reasonable that not all individuals will register AF at the hospitals upon returning to Denmark.

In this study magnesium in drinking water at water supply area level was used as a proxy for the actual individual magnesium intake. In view of this, a common limitation in studies on drinking water and disease is the ecological nature of exposure which introduces a possibility of misclassification. In our register-based study, we were not able to include individual intake of magnesium from drinking water. Furthermore, the frequency of magnesium measurements at each waterworks ranges [34] and the water supply areas are assumed constant in time. No statistically significant difference was found between magnesium concentration measured at household and at waterworks, respectively [34]. The regional concentration trend in magnesium in drinking water increases the likelihood for consuming the same magnesium concentration in drinking water at e.g. work compared to at the residential address. The two sensitivity analyses, one with only addresses within water supply areas with at least one water sample per every second year, and one with only addresses within water supply areas with constant concentration in time, did not change the results. The data file with the geographical distribution of water supply areas was created in 2013–2014 [50], with minor updates in 2017 [34], and are assumed stationary (2002–2015). Water supply areas covering more than one waterworks, might have been divided into smaller water supply areas prior to 2013, and magnesium concentration in drinking water might therefore have differed within the water supply area which has not been accounted for in this study. This might have led to misclassification of magnesium exposure, especially for years before and after the period where the water supply area data file was created. However, the patterns of magnesium concentration in drinking water make it likely that neighboring waterworks have similar magnesium levels, reducing the risk of misclassification.

Altogether, exposure misclassification in our study is expected to be independent of the incidence of AF and the effect on the result would be towards no association between magnesium in drinking water and incidence of AF.

AF is expected to be underestimated in the population [1], since AF may exist subclinical asymptomatic. Thereby, only the more severe or persistent AF is registered in the health registers. If the geographical variation in subclinical asymptomatic AF correlates with the magnesium exposure groups, this could potentially introduce a bias. Furthermore, little is known about the association between magnesium in drinking water and the latency time to AF. It has been suggested that it is the present magnesium exposure that is relevant for an effect [46]. If a more instant response is expected, an error may have been introduced by calculating a 5-year mean magnesium concentration. However, by repeating the analyses using both a 1-year and 2-year weighted mean, we have accounted for the possibility of a short-term effect. On the other hand, if the development of AF is expected to be caused by long-term exposure to low level of magnesium in drinking water, the analyses where only the first exposure group was included may be more relevant.

When using administratively collected data, it is a common limitation that not all desired confounders are available in the registers [49]. It seems unlikely that individual risk factors for incident AF effect the magnesium concentration in drinking water. To support this, no strong effect on the results was observed, when adjusting for socioeconomic position. However, the larger regional difference in magnesium concentration in drinking water may lead to the possibility of correlation between magnesium in drinking water at water supply area level and possible AF risk factors with similar geographical patterns at a regional scale. This could be an explanation for the findings of an overall positive association between magnesium in drinking water and increasing incidence of AF. While individual risk factors are important to consider, the place where each individual lives also has some significance [51]. Geographical variations in contextual risk factors, e.g. environmental exposures or regional health service structures, could correlate with the geographical variation in magnesium exposure. Furthermore, Dummer [52] has highlighted the importance of understanding the geographical variation in health services and environmental exposures and their interrelations, when working with health-related risk exposures. Our results suggest that there are risk factors operating at a regional level that effect our results, since an inverse rather than a positive association between magnesium in drinking water and incident AF was found, when restricting the analysis to one administrative region.

Magnesium in drinking water may correlate with other inorganic chemical compounds in drinking water, and it is therefore plausible that these other compounds partly explain the results. In Denmark, magnesium in drinking water correlates with e.g. calcium [34]. However, it has been concluded in several studies that the association with CVD is more likely to be related to magnesium than calcium in drinking water [15, 46]. Other possible chemical compounds that may be associated with AF risk factors and correlate with magnesium in drinking water include e.g. sodium. However, compared to the dietary sodium intake, the fraction from drinking water is small [53].

It has been argued, that the effect of magnesium in drinking water may only be found in populations where the dietary magnesium intake is insufficient [54], though by including a large population, we would expect to detect even a small effect, if any. In the age-stratified analyses, we saw a different association in the age group ≥80 years, which is considered as a more vulnerable group in relation to magnesium deficiency [55].

## Conclusion

There might be a small beneficial effect of an increase in magnesium level in drinking water up to 10 mg/L, though an unexpected overall trend indicates a small positive association between magnesium in drinking water and AF risk. The results were consistent across changes in exposure categories. Results were similar in men and women, while the strongest positive associations were observed among those aged ≥80 years and in the lowest education group. An inverse association was found for the highest education group. The small positive association was not seen when restricting the analysis to the Region of Southern Denmark. The different associations observed for specific subgroups suggest the potential influence of unaccounted factors in the association between magnesium in drinking water and AF, particularly in vulnerable populations. Future research on magnesium in groundwater based drinking water and CVDs needs to focus on contextual risk factors, especially those potentially correlating with magnesium in drinking water.

## Supplementary Information

**Additional file 1.** Present incidence rate ratios (IRRs) of atrial fibrillation and confidence intervals (CI) presented in forest plots for the ad-hoc analysis including only the Region of Southern Denmark. The lowest magnesium (Mg) exposure group is the reference group in the analysis. The analysis is adjusted for age, sex and socioeconomic position.

## Data Availability

The data that support the findings of this study are available from the National Health Authority and Statistics Denmark, but restrictions apply to the availability of these data, which were used under license for the current study, and so are not publicly available.

## Abbreviations

AF: Atrial fibrillation
CVD: cardiovascular disease ()
CI: confidence interval
IR: incidence rate
HR: hazard ratio
IRRs: incidence rate ratios
PIN: personal identification number
RDI: recommended daily intake
TWA: Time-weighted average
WHO: World Health Organization
