# Supplementary material for: Association between magnesium in drinking water and atrial fibrillation incidence: a nationwide population-based cohort study, 2002–2015
Source: Environ Health. 2021 Dec 15;20:126. doi: 10.1186/s12940-021-00813-z (PMC8672465; doi:10.1186/s12940-021-00813-z)
Supplement: Supplementary file 1 — Additional file 1. Present incidence rate ratios (IRRs) of atrial fibrillation and confidence intervals (CI) presented in forest plots for the ad-hoc analysis including only the Region of Southern Denmark. The lowest magnesium (Mg) exposure group is the reference group in the analysis. The analysis is adjusted for age, sex and socioeconomic position. [file 12940_2021_813_MOESM1_ESM.pdf]

Additional file 1

Analysis<br>Mg exposure [mg/L]	IRR (95% CI)	P-value	Person years (PY)<br>(% of total PY)
**Region of Southern Denmark**			**5,182,838**
0.1-5	1 (ref)		(13.9)
>5-10	0.95 (0.91; 0.95)	0.005	(40.9)
>10-15	0.93 (0.89; 0.97)	0.000	(39.0)
>15-20	0.96 (0.90; 1.02)	0.200	(5.0)
>20-62	0.83 (0.75; 0.93)	0.001	(1.3)

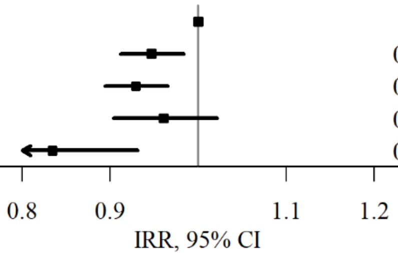

Additional file 1: Incidence rate ratios (IRRs) of atrial fibrillation and confidence intervals (CI) presented in forest plots for the ad-hoc analysis including only the Region of Southern Denmark. The lowest magnesium (Mg) exposure group is the reference group in the analysis. The analysis is adjusted for age, sex and socioeconomic position.
